# Supplementary material for: Does Physical Activity in Natural Outdoor Environments Improve Wellbeing? A Meta-Analysis
Source: Sports (Basel). 2022 Jun 29;10(7):103. doi: 10.3390/sports10070103 (PMC9315957; doi:10.3390/sports10070103)
Supplement: Supplementary file 1 [file sports-10-00103-s001.zip › sports-1744793-supplementary.pdf]

Table S1: PRISMA Checklist

Section and Topic	Item #	Checklist item	Location where item is reported
**TITLE**			
Title	1	Identify the report as a systematic review.	Page 1
**ABSTRACT**			
Abstract	2	See the PRISMA 2020 for Abstracts checklist.	Page 1
**INTRODUCTION**			
Rationale	3	Describe the rationale for the review in the context of existing knowledge.	Page 2
Objectives	4	Provide an explicit statement of the objective(s) or question(s) the review addresses.	Page 2
**METHODS**			
Eligibility criteria	5	Specify the inclusion and exclusion criteria for the review and how studies were grouped for the syntheses.	Page 2; Supplemental Table 2
Information sources	6	Specify all databases, registers, websites, organisations, reference lists and other sources searched or consulted to identify studies. Specify the date when each source was last searched or consulted.	Page 2
Search strategy	7	Present the full search strategies for all databases, registers and websites, including any filters and limits used.	Page 2
Selection process	8	Specify the methods used to decide whether a study met the inclusion criteria of the review, including how many reviewers screened each record and each report retrieved, whether they worked independently, and if applicable, details of automation tools used in the process.	Page 3
Data collection process	9	Specify the methods used to collect data from reports, including how many reviewers collected data from each report, whether they worked independently, any processes for obtaining or confirming data from study investigators, and if applicable, details of automation tools used in the process.	Page 3
Data items	10a	List and define all outcomes for which data were sought. Specify whether all results that were compatible with each outcome domain in each study were sought (e.g. for all measures, time points, analyses), and if not, the methods used to decide which results to collect.	Page 2
	10b	List and define all other variables for which data were sought (e.g. participant and intervention characteristics, funding sources). Describe any assumptions made about any missing or unclear information.	Page 2
Study risk of bias assessment	11	Specify the methods used to assess risk of bias in the included studies, including details of the tool(s) used, how many reviewers assessed each study and whether they worked independently, and if applicable, details of automation tools used in the process.	Page 2
Effect measures	12	Specify for each outcome the effect measure(s) (e.g. risk ratio, mean difference) used in the synthesis or presentation of results.	Page 3
Synthesis	13a	Describe the processes used to decide which studies were eligible for each synthesis (e.g. tabulating the study	Page 4; Supplemental

Section and Topic	Item #	Checklist item	Location where item is reported
methods		intervention characteristics and comparing against the planned groups for each synthesis (item #5)).	Table 2
	13b	Describe any methods required to prepare the data for presentation or synthesis, such as handling of missing summary statistics, or data conversions.	Page 3
	13c	Describe any methods used to tabulate or visually display results of individual studies and syntheses.	Page 3
	13d	Describe any methods used to synthesize results and provide a rationale for the choice(s). If meta-analysis was performed, describe the model(s), method(s) to identify the presence and extent of statistical heterogeneity, and software package(s) used.	Page 4
	13e	Describe any methods used to explore possible causes of heterogeneity among study results (e.g. subgroup analysis, meta-regression).	Page 4
	13f	Describe any sensitivity analyses conducted to assess robustness of the synthesized results.	Page 3/4
Reporting bias assessment	14	Describe any methods used to assess risk of bias due to missing results in a synthesis (arising from reporting biases).	NR
Certainty assessment	15	Describe any methods used to assess certainty (or confidence) in the body of evidence for an outcome.	Page 3
**RESULTS**			
Study selection	16a	Describe the results of the search and selection process, from the number of records identified in the search to the number of studies included in the review, ideally using a flow diagram.	Page 3, Supplemental Figure 1
	16b	Cite studies that might appear to meet the inclusion criteria, but which were excluded, and explain why they were excluded.	NR
Study characteristics	17	Cite each included study and present its characteristics	Supplemental Tables 2, 4
Risk of bias in studies	18	Present assessments of risk of bias for each included study.	Pages 3 – 5
Results of individual studies	19	For all outcomes, present, for each study: (a) summary statistics for each group (where appropriate) and (b) an effect estimate and its precision (e.g. confidence/credible interval), ideally using structured tables or plots.	Pages 4 – 6
Results of syntheses	20a	For each synthesis, briefly summarise the characteristics and risk of bias among contributing studies.	Pages 3, 5
	20b	Present results of all statistical syntheses conducted. If meta-analysis was done, present for each the summary estimate and its precision (e.g. confidence/credible interval) and measures of statistical heterogeneity. If comparing groups, describe the direction of the effect.	Pages 3-6
	20c	Present results of all investigations of possible causes of heterogeneity among study results.	Pages 3-6
	20d	Present results of all sensitivity analyses conducted to assess the robustness of the synthesized results.	Pages 3-6

Section and Topic	Item #	Checklist item	Location where item is reported
Reporting biases	21	Present assessments of risk of bias due to missing results (arising from reporting biases) for each synthesis assessed.	NR
Certainty of evidence	22	Present assessments of certainty (or confidence) in the body of evidence for each outcome assessed.	Pages 4, Supplemental Table 3 and 5
**DISCUSSION**			
Discussion	23a	Provide a general interpretation of the results in the context of other evidence.	Pages 6-7
	23b	Discuss any limitations of the evidence included in the review.	Pages 7-8
	23c	Discuss any limitations of the review processes used.	Page 7
	23d	Discuss implications of the results for practice, policy, and future research.	Pages 7
**OTHER INFORMATION**			
Registration and protocol	24a	Provide registration information for the review, including register name and registration number, or state that the review was not registered.	Page 2
	24b	Indicate where the review protocol can be accessed, or state that a protocol was not prepared.	Page 2
	24c	Describe and explain any amendments to information provided at registration or in the protocol.	NR
Support	25	Describe sources of financial or non-financial support for the review, and the role of the funders or sponsors in the review.	Page 8
Competing interests	26	Declare any competing interests of review authors.	Page 8
Availability of data, code and other materials	27	Report which of the following are publicly available and where they can be found template data collection forms; data extracted from included studies; data used for all analyses; analytic code; any other materials used in the review.	Page 8

Table S2: PICOS Framework: Review Eligibility

PICOS Item	Inclusion Criteria	Exclusion Criteria
Population	Adults (mean sample age ≥ 18 years of age) without reported health condition.	Children (mean sample age < 18 years of age) and special populations (e.g., clinical populations).
Intervention	Physical activity (PA) interventions in the natural outdoor environment (NOE). The interventions can be in the form of any type of physical activity or exercise, of any duration, frequency and intensity of physical activity. At least one condition in the study must pursue PA in the NOE. Should an intervention include multiple manipulations (e.g. PA + health education, PA + cognitive tasks) only condition(s) without the added manipulation will be included in the analysis.	Interventions in non-natural outdoor environments/ in-built environments, interventions occurring indoors with virtual reality simulations/ projections of the natural environment/ photographs of the natural environment, interventions including some other component (e.g. health education or any other manipulation) for all conditions or has no condition for PA in the NOE.
Comparison	Where possible (based on study design) a physical activity group located indoors will be included as comparison. As quasi-experimental designs will also be included, not all studies will have a comparison/control but will be included in the primary analysis. The indoor comparison can be in the form of any duration, intensity or type so long as the physical environment in which it occurs is indoors only.	Indoor physical activity intervention with any kind of manipulation (projections of virtual reality (VR), photos, images, videos of green space or audio stimuli). And/or, any comparison to environments not consistent with the natural outdoor environment (e.g. urban environments marked by streets, intersections, man-made structures). Any sedentary/rest control groups.

Outcomes	Changes in mean well-being scores (i.e., pre-post) across at least one well-being indicator consistent with Subjective Well-Being (Diener, 1984), Psychological Well-Being (Ryff, 1989) and/or Flourishing (Huppert & So, 2013)	Measures of well-being inconsistent with the above-mentioned conceptual approaches to well-being.
Studies	Randomized controlled trials, controlled trials, quasi-experimental designs.	Qualitative studies, review studies, studies not published in English, dissertations, conference proceedings, thesis documents

Table S3: Study & Sample Characteristics for Primary Purpose

Study, Year	*n*	Study Design	Outcome(s)	PA type	PA (Mins)	PA Intensity	NOE Type
Fuegen & Breitenbecker, 2018	41	Quasi-Experimental	Pos. Affect Vitality	Walk	15	Low	Urban Greenspace
Pasanen et al. 2018(a)	41	Quasi - Experimental	Pos. Affect	Walk	103	Self-Selected	Mixed
Pasanen et al. 2018 (b)	40	Quasi-Experimental	Pos. Affect	Walk	120	Self-Selected	Mixed
Pretty et al. 2007	263	Quasi-Experimental	Self Esteem Vitality	Mixed	60	Self-Selected	Mixed
Diessner et al. 2015	21	Quasi-Experimental	Engagement	Walk	10	NR	Urban Greenspace
Harte & Eifert, 1995	10	Quasi-Experimental	Vitality	Running	45	Vigorous	Urban Greenspace
Geniole et al. 2016	31	Quasi-Experimental	Pos. Affect	Walk	15	Moderate	Urban Greenspace
Bodin et al. 2003	12	Quasi-Experimental	Vitality	Running	60	Moderate	Mixed
Johansson et al. 2011	20	Quasi-Experimental	Vitality Engagement	Walk	40	Moderate	Urban Greenspace
Crust et al. 2013	83	Quasi-Experimental	Pos. Affect Self Esteem	Walk	60	Low	Mixed
Rogerson et al. 2016	24	Pre-Post Matched Groups	Vitality	Cycling	15	Moderate	Urban Greenspace
Byrka & Ryczko, 2018	28	Quasi-Experimental	Vitality Engagement	Dance	40	NR	Urban Greenspace
Niedermeier et al. 2017	42	Pre-Post Matched Groups	Pos. Affect Pos. Emotion	Hike	230	Self-Selected	Woodland/ Forest
Turner et al. 2017	22	Pre-Post Matched Groups	Pos. Affect Vitality	Running	NR	MVPA	Woodland/ Forest

Focht, 2009	35	Pre-Post Matched Groups	Pos. Affect Vitality Engagement	Walk	10	Self-Selected	Urban Greenspace
Flowers et al. 2018	15	Pre-Post Matched Groups	Vitality Self Esteem	Cycling	15	Moderate	Urban Greenspace
Ryan et al., 2009	40	Modified Randomized Experiment	Vitality	Walk	15	NR	Urban Greenspace
Ekkekakis et al. 2008	6	Quasi-Experimental	Pos. Affect	Walk	15	Moderate	Bluespace/ Freshwater
Ekkekakis et al., 2000	26	Quasi-Experimental	Pos. Affect	Walk	10	Self-Selected	Urban Greenspace

Note. n = sample size, PA = physical activity, NOE= Natural Outdoor Environment, NR = not reported, Pos. Affect = Positive Affect, Pos. Emotion = Positive Emotion, Sessions refers to number of sessions of physical activity, MVPA = moderate to vigorous physical activity.

Table S4: Effect size by Measure and Study for PA in the NOE on Wellbeing

Measure	*Study*	*ES*	*SE*	*Variance*	*p-value*	*95% CI [Lb, Ub]*
Engagement						
	Diessner, 2015	-0.10	0.22	0.05	0.63	[-0.53, 0.32]
	Johansson, 2011	0.31	0.23	0.05	0.18	[-0.14, 0.76]
	Byrka, 2018	0.54	0.25	0.06	0.03	[0.05, 1.02]
	Focht, 2009	0.47	0.18	0.03	0.01	[0.12, 0.82]
Total		0.30	0.14	0.02	0.03	[0.02, 0.59]
Pos. Affect						
	Fuegen, 2018	0.19	0.16	0.02	0.22	[-0.12, 0.50]
	Pasanen, 2018(a)	0.93	0.19	0.03	0.00	[0.56, 1.30]
	Pasanen, 2018(b)	0.88	0.19	0.03	0.00	[0.52, 1.25]
	Geniole, 2016	0.59	0.25	0.06	0.02	[0.10, 1.08]
	Crust, 2013	0.15	0.11	0.01	0.17	[-0.07, 0.37]
	Niedermeier, 2017	0.92	0.20	0.04	0.00	[0.51, 1.32]
	Turner, 2017	-0.35	0.22	0.05	0.11	[-0.78, 0.08]
	Focht, 2009	0.98	0.21	0.04	0.00	[0.58, 1.38]
	Ekkekakis, 2008	0.50	0.41	0.17	0.22	[-0.30,1.30]
	Ekkekakis, 2000	0.86	0.20	0.04	0.00	[0.47, 1.25]
Total		0.56	0.14	0.02	0.00	[0.28, 0.84]
Self Esteem						
	Pretty, 2007	0.31	0.06	0.00	0.00	[0.18, 0.43]
	Crust, 2013	0.51	0.12	0.01	0.00	[0.28, 0.74]
	Flowers, 2018	0.89	0.30	0.09	0.00	[0.29, 1.48]
Total		0.45	0.12	0.01	0.00	[0.22, 0.69]
Vitality						
	Fuegen, 2018	0.63	0.17	0.03	0.00	[0.29, 0.96]
	Pretty, 2007	0.03	0.06	0.00	0.60	[-0.09, 0.15]
	Harte, 1995	-0.82	0.37	0.13	0.02	[-1.54, -0.11]
	Bodin, 2003	1.17	0.38	0.14	0.00	[0.44, 1.91]
	Johansson, 2011	0.47	0.24	0.06	0.05	[0.01, 0.93]
	Rogerson, 2016	0.15	0.21	0.04	0.46	[-0.25, 0.56]
	Byrka, 2018	1.33	0.33	0.11	0.00	[0.68, 1.97]
	Turner, 2017	0.91	0.25	0.06	0.00	[0.42, 1.41]
	Focht, 2009	0.96	0.20	0.04	0.00	[0.56, 1.36]
	Flowers, 2018	0.59	0.28	0.08	0.03	[0.04, 1.14]
	Ryan, 2009	0.43	0.17	0.03	0.01	[0.10, 0.75]
Total		0.52	0.15	0.02	0.00	[0.22, 0.82]

Note. ES = standardized mean difference (Cohen's *d*); *SE* = standard error; *95% CI [Lb, Ub]* = lower and upper bounds of the 95% confidence interval. Positive emotion was not included in table as it was only measured in one study. Only the last name of the primary author and year of publication was included under study name.

Table S5: Study and Sample Characteristics for PA in the NOE and Indoor Comparison on Wellbeing

Study, Year	Sample Ind./NOE	Outcome Measure(s)	PA Type	PA Mins.	PA Intensity	NOE Type
Rogerson et al. 2016	24/24	Vitality	Cycle	15	Moderate	Urban Greenspace
Niedermeier et al. 2017	42/42	Pos. Affect<br>Pos. Emotion	Hike	230	Self-Selected	Woodland/ Forest
Turner et al. 2017	22/22	Pos. Affect<br>Vitality	Run	NR	MVPA	Woodland/ Forest
Focht, 2009	35/35	Engagement<br>Pos. Affect<br>Vitality	Walk	10	Self-Selected	Urban Greenspace
Flowers et al. 2018	15/15	Self Esteem<br>Vitality	Cycle	15	Moderate	Urban Greenspace

Note. Ind. = indoor. PA = physical activity, NR = not reported, Pos. Affect = Positive Affect, PA mins = duration of PA session in minutes, MVPA= moderate to vigorous physical activity. All studies were coded as having one session indoors and one session in the NOE. All studies were coded as pre/post matched groups comparison study design.

Table S6: Effect size by Measure and Study for PA in the NOE and Indoor Comparison on Wellbeing

Measure	*Study*	*ES* *NOE/Indoor*	*Variance* *NOE/Indoor*	*p-Value* *NOE/Indoor*	*95% CI [Lb, Ub]* *NOE/Indoor*
Pos. Affect					
	Focht, 2009	0.98/0.73	0.04/0.04	0.00/0.00	[0.58, 1.38]/[0.36, 1.54]
	Niedermeier, 2017	0.15/0.18	0.03/0.02	0.00/0.23	[0.53, 1.22]/[-0.12, 0.65]
	Turner, 2017	-0.35/-0.39	0.05/0.05	0.11/0.08	[-0.78, 0.08]/[-0.82, 0.05]
Total		0.51/0.18	0.12/0.12	0.15/0.60	[-0.18, 1.20][-0.50, 0.86]
Vitality					
	Flowers, 2018	0.59/0.14	0.08/0.07	0.03/0.58	[0.04, 1.14]/[-0.37, 0.65]
	Focht, 2009	0.96/0.95	0.04/0.04	0.00/0.00	[0.56, 1.36]/[0.55, 1.35]
	Rogerson, 2016	0.15/0.19	0.04/0.04	0.46/0.36	[-0.25, 0.56]/[-0.22, 0.59]
	Turner, 2017	0.91/0.47	0.06/0.05	0.00/0.04	[0.42, 1.41]/[0.03, 0.91]
Total		0.65/0.45	0.04/0.04	0.00/0.02	[0.26, 1.04]/[0.06, 0.84]

Note. ES = standardized mean difference (Cohen's *d*); *SE* = standard error; *95% CI [Lb, Ub]* = lower and upper bounds of the 95% confidence interval. Only the last name of the primary author and year of publication was included under study name.

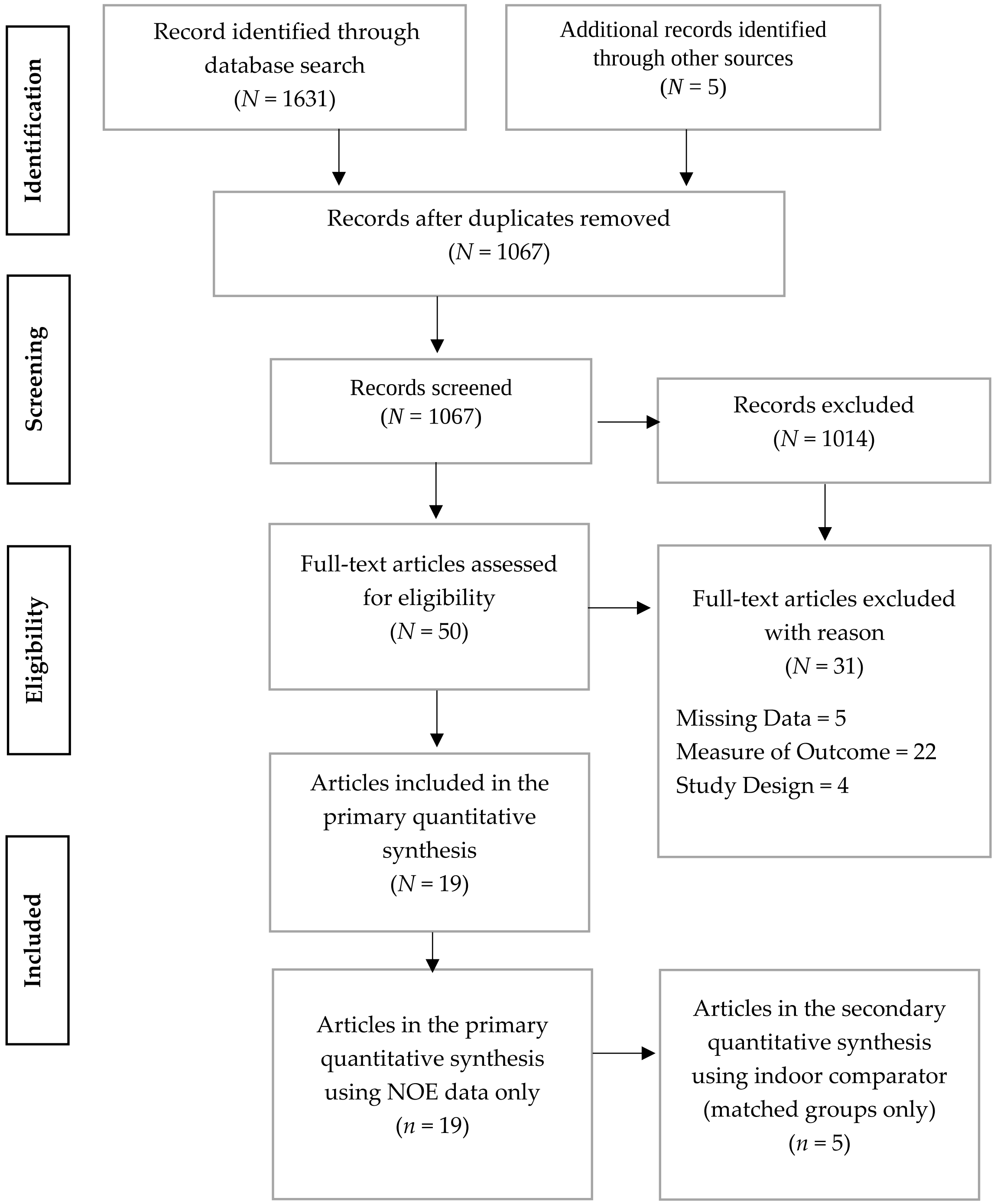

Figure S1. PRISMA flow diagram
